# Saikosaponin D Mitigates Radioresistance in Triple-Negative Breast Cancer by Inducing MRE11 De-Lactylation via HIF1α/HDAC5 Pathway

**DOI:** 10.7150/thno.113517

**Published:** 2025-08-11

**Authors:** Jingyi Li, Liang Feng, Lei Zhang, Weiguo Zhang, Yanting Zhang, Xiaoqin Liu, Qianqian Cai, Weiming Zhao, Gang Huang, Changlian Lu

**Affiliations:** 1 Shanghai Key Laboratory of Molecular Imaging, Jiading District Central Hospital Affiliated Shanghai University of Medicine and Health Sciences, Shanghai, China.; 2 Heilongjiang University of Traditional Chinese Medicine, Harbin, Heilongjiang, China.; 3 School of Pharmacy, Shanghai University of Medicine and Health Sciences, Shanghai, China.; 4 Department of Radiation Oncology, Renji Hospital, School of Medicine, Shanghai Jiao Tong University, Shanghai 200127, China.

**Keywords:** Triple-negative breast cancer (TNBC), Lactylation, Saikosaponin D (SSD), histone deacetylase (HDAC) 5, radioresistance

## Abstract

**Background:** Triple-negative breast cancer (TNBC), the most aggressive breast cancer subtype, exhibits poor prognosis due to radiotherapy resistance. However, the underlying mechanisms and effective therapeutic agents remain elusive.

**Methods:** We employed lactate/oxamate to assess DNA damage/repair in irradiated TNBC cell lines. Lentiviral vectors for MRE11/HDAC5 constructs and shRNA were used to explore lactylation via Western blot/Co-IP. TCGA data mining, tissue microarrays, proteomics-MS, and gene expression profiling were used to dissect Saikosaponin D (SSD)'s radiosensitizing mechanisms. Promoter luciferase assays and ChIP-qPCR were performed to map SSD-induced HIF1α binding sites on the HDAC5 promoter.

**Results:** Elevated endogenous lactate in radioresistant TNBC cells promoted DNA repair via MRE11 Lys^673^ lactylation, a critical modification conferring radioresistance. HDAC5 was identified as the key delactylase for MRE11 Lys^673,^ validated by HADDOCK docking (hydrogen bond between MRE11 Lys^673^ and HDAC5 Ser^18^) and Co-IP (HDAC5 overexpression reduced K^673^ lactylation). TCGA and clinical tissue microarrays confirmed HDAC5 downregulation in TNBC. SSD inhibits the malignant phenotype of TNBC and enhances radiotherapy efficacy by inhibition on MRE11 lactylation via upregulating HDAC5. Mechanistically, SSD upregulated HIF1α to bind the HDAC5 promoter (-342bp to -20bp region) to activate its expression.

**Conclusion:** Lactate-driven MRE11 Lys^673^ lactylation mediates radioresistance, while SSD reverses this via HIF1α/HDAC5 axis activation. Our findings identify SSD as a radiosensitizer and HDAC5/MRE11 as potential therapeutic targets for TNBC.

## Introduction

In recent years, global cancer rates have risen, with breast cancer now having surpassed lung cancer as the most common type. Triple-negative breast cancer (TNBC), characterized by the absence of estrogen, progesterone, and HER2 receptors, accounts for about 17% of breast cancer cases 1. While early symptoms are often subtle, TNBC is highly aggressive, with high metastasis rates and recurrence rates, poor prognosis, and short survival time 2. Radiotherapy plays a key role in improving local tumor control, enhancing treatment efficacy, and prolonging survival, especially in early and advanced stages 3, 4. However, resistance to radiotherapy often leads to tumor persistence or recurrence, negatively impacting prognosis 5. Despite efforts to understand and overcome this resistance, the underlying molecular mechanisms remain unclear.

Metabolic reprogramming and genomic instability are two key characteristics of cancer cells 6-8. Enhanced glycolysis in tumor cells leads to lactate accumulation. Lactate mediates a novel post-translational modification called lactylation (Kla), which plays a role in regulating tumor progression, immune inflammation, cardiovascular diseases, neurological disorders, and metabolic diseases through various signaling pathways and key molecules 6, 9-19. Recent studies have shown that chemotherapy-induced DNA double-strand breaks (DSBs) are repaired through lactylation, contributing to tumor resistance to chemotherapy. This has led to the proposal that lactylation inhibitors could serve as a novel therapeutic strategy for overcoming chemotherapy resistance in tumors 20, 21.

MRE11, a central component of the MRN complex involved in DNA repair, possesses single-strand endonuclease activity and double-strand-specific 3'-5' exonuclease activity 22. Upon DSBs, MRE11 is recruited to the damage sites and, in collaboration with RBBP8/CtIP, initiates end resection, generating single-stranded DNA gaps through 3' to 5' exonucleolytic degradation. This process provides entry sites for EXO1- and DNA2- mediated long-range 5' to 3' resection, which is essential for single-strand invasion and recombination 23. MRE11's activity is regulated by various post-translational modifications, including phosphorylation, ubiquitination, and lactylation 24-27. Phosphorylation at Ser^676^ and Ser^678^ by ATM in response to DNA damage promotes MRE11 activity, while phosphorylation at Ser^649^ by PLK1 primes further phosphorylation at Ser^688^ by CK2, inhibiting MRN complex recruitment to DNA damage sites. Recent findings suggest that lactylation of MRE11 at Lys^673^ by CREBBP/CBP in response to DNA damage enhances DNA binding and MRE11 activity 21. However, the role of MRE11 lactylation in the Warburg effect and its potential involvement in radiotherapy response remain undefined.

Lactylation is a dynamic and reversible modification catalyzed by specific enzymes. The histone deacetylase (HDAC) family comprises enzymes responsible for hydrolyzing modification groups on histone and non-histone substrates. These enzymes are crucial for maintaining chromatin state and regulating downstream signaling pathways 28. The HDAC family is categorized into four classes: Class I (HDAC1-3, 8), Class II (HDAC4, 5, 7, 9), Class III (SIRT1-7), and Class IV (HDAC11). It has been reported that HDAC1 and 3 are the primary "erasers" of lysine lactylation on histones (H3K18 and H4K5), while SIRT1 and SIRT3 are potential lactylation erasers for non-histone proteins (e.g., PKM2, ENO1) 20, 29-31. Huang et al. found that the Class III HDAC member SIRT2 mediates the removal of lactylation at METTL16-K229 and inhibits METTL16 activity 32. Combination of the SIRT2-specific inhibitor AGK2 with cuproptosis (copper-induced cell death) potently enhances gastric cancer treatment outcomes. Combining the SIRT2-specific inhibitor AGK2 with copper-induced cell death significantly. It is worth noting that, unlike other HDAC classes, Class II HDAC member HDAC5 is typically downregulated or deleted in various human cancers, with a significant correlation to patient prognosis 33. However, to date, the involvement of HDAC5 in lactylation has not been investigated.

This study addresses radioresistance in TNBC treatment and identifies lactate accumulation as a key driver of DNA repair-mediated radiotherapy resistance. Lactate induces lactylation of the DNA repair protein MRE11 at Lys^673^, establishing this modification as a critical mediator of radioresistance. We further discovered that lactyl groups on MRE11 Lys^673^ can be removed by HDAC5, identifying HDAC5 as a novel "eraser" of lactylation. Finally, Saikosaponin D (SSD) activates the HIF1α pathway and HIF1α binds to a specific region of the HDAC5 promoter, enhancing its transcriptional activity. This leads to HDAC5-mediated de-lactylation of MRE11, thereby reversing radiotherapy resistance. Collectively, this research explores the molecular mechanisms of radioresistance and identifies potential therapeutic targets, demonstrating that SSD can mitigate radioresistance, providing experimental support for the development of MRE11/HDAC5-targeted therapies and SSD-based drugs in the future.

## Materials and Methods

### Cell culture and treatment

Human TNBC cell lines MDA-MB-231 and MDA-MB-468 were obtained from ATCC (Manassas, VA, USA). Cells were cultured in DMEM supplemented with 10% fetal bovine serum (FBS; #FBSAD-01011, Santa Clara Biotech) at 37 °C in ambient air. For experimental treatments, cells were exposed to sodium lactate (Cat #S108838, Shanghai Aladdin, purity ≥ 98%), sodium oxamate (Cat#3893, APExBIO, purity >99.5%), or Saikosaponin D (SSD, Cat #S5454, Selleckchem, purity > 90%) for 24 h. Mycoplasma contamination was monitored every 2 weeks using a mycoplasma detection kit (Yeasen). Endogenous lactate levels were measured as previously described 34. Ionizing radiation (IR) was delivered using a 6 MV X-ray linear accelerator (Primus, Siemens AG, Germany) at a dose rate of 200 MU/min, with doses ranging from 0 to 16 Gy.

### Vector construction, transfection and reverse transcription quantitative real-time PCR (RT-qPCR)

For the construction of wild-type (WT) and mutant MRE11 plasmids, the wild-type sequence of Homo sapiens MRE11 (NM_005591.4) and the mutant MRE11K673R (encoding a codon change from AAG to CGT) were PCR amplified and inserted into the PLVX-IRES-PuroR vector using EcoRI/XhoI recognition sites, with the addition of a 3Flag coding sequence. For HDAC5 overexpression, the coding sequence of HDAC5 (NM_005474.5) was cloned into the PGMLV-CMV-MCS-HA-PGK-puro vector using XhoI/EcoRI restriction sites. The plasmid was extracted and used to transfect MDA-MB-231 cells to research the interaction and effects of HDAC5 with MRE11. For the construction of the H_HDAC5 shRNA interference vector, the target sequences were 5′TTCTCCGAACGTGTCACGT for shNC, 5′GCAAGGATGGGACTGTTATTA for shHDAC5#1, 5′GCTCAAGAATGGATTTGCCAT for shHDAC5#2, and 5′GCTAGAGAAAGTCATCGAGAT for shHDAC5#3. The vector was linearized using restriction enzymes, and the target shRNA sequence was inserted to construct the H_HDAC5-shRNA (PGMLV-Puro) vector. The plasmid was then transfected into HEK-293T cells to produce lentivirus particles, which were subsequently used to transfect MDA-MB-231 cells. RT-qPCR and WB were performed to verify the knockdown efficiency of each shRNA 34, 35 and sequences of primer for RT-qPCR are listed in Table S1.

### Immunofluorescence detection of γH2AX foci

MDA-MB-231 and MDA-MB-468 cells in the logarithmic growth phase were seeded in Lab-Tek II chambers at 1 × 10^5^ cells per well and cultured to 60% confluence. Cells were treated with lactate, Oxamate, and SSD at indicated concentration for 24 h and irradiated with 4 Gy. At 0 h, 8 h, and 24 h, cells were fixed, permeabilized, and incubated with γH2AX antibody (Cat#AF7092, 1:200, Beyotime) overnight. After secondary antibody incubation, cells were stained with DAPI and analyzed by confocal microscopy for γH2AX foci formation.

### Comet assay for DNA damage detection

Cells were treated with lactate, Oxamate, and SSD for 24 h. After 4 Gy irradiation, cells were collected at 0 h, 8 h, and 24 h, counted to 1 × 10^6^ cells/mL, and applied to pre-warmed slides with 30 µL 1% agarose gel. After solidifying at 4 °C for 10 min, slides were incubated overnight in lysis buffer at 4 °C. The next day, slides were submerged in electrophoresis buffer for 1 h, electrophoresed at 25 V for 30 min. 20 µL Propidium Iodide Solution was added to stain for 10-20 min, and DNA damage was observed and photographed under a confocal microscope (Leica CTS-SP8 laser, Leica Microsystems). Comet images were analyzed using CASP 1.2.2 software, Tail DNA (%) = 100 × Tail DNA Intensity / Cell DNA Intensity.

### Apoptosis and cloning formation

Apoptosis analysis and cloning formation were administrated as previously 34, 35. For colony survival fraction analysis, 2,000 cells/well were seeded in 6-well plates and treated with control, 20 mM lactate, or 20 mM Oxamate. After 24 h, cells were irradiated (0-10 Gy), cultured for 2 weeks, and stained with crystal violet. For the cells without irradiation in each group, the number of clones was taken as 100%, and the percentage of cell clones under different irradiation intensities was calculated as the survival fraction.

### Western blotting (WB) and Co-immunoprecipitation (Co-IP)

WB was performed as described previously 35, using the following antibodies: β-actin (Cat#66009-1, 1:10000, Proteintech), HDAC5 (Cat#16166-1, 1:1000, Proteintech), MRE11 (Cat#4895, 1:1000, Cell Signaling Technology), L-Lactyl Lysine (Cat#PTM-1401RM, 1:500, PTM Biolab); MRE11 K673lac specific antibody (Cat#CP0326, PTM Biolab), NBS1(Cat#55025-1, 1:1000, Proteintech), Anti-Acetyllysine Rabbit mAb (Cat#105RM, PTM Biolab), RAD50 (Cat#AF7857, Beyotime, China), HIF1α (Cat#20960-1, Beyotime, China), β-Tubulin (Cat# AF1216 1:5000, Beyotime). Chemiluminescent detection of membranes was performed using a Tanon 5200 imaging system (Tanon Science & Technology, Shanghai, China).

Co-IP was carried out with the Immunoprecipitation Kit with Protein A/G Magnetic Beads (Selleck, B23201). Cell lysis was performed on ice using a buffer optimized for immunoprecipitation (20 mM Tris-HCl, pH 7.5; 150 mM NaCl; 1% Triton X-100) supplemented with sodium pyrophosphate, β-glycerophosphate, EDTA, sodium orthovanadate, leupeptin, and other protease and phosphatase inhibitors. Antibodies incubated with beads overnight at 4 °C, including anti-Flag-tag (Cat#F1804, 1:100, Sigma); anti-HA-tag (Cat#3724, 1:100, Cell Signaling Technology). Following repeated washes, bead-bound proteins were eluted in 1×SDS-PAGE Protein Loading Buffer (LT101, Epizyme), denatured at 100 °C for 8-10 min, and subjected to WB.

### Analysis of drug synergistic effects

To determine the synergistic effects between SSD and radiotherapy, TNBC cells were treated with graded concentrations of SSD and varying doses of radiation. The Combination Index (CI) values for the two therapeutic agents were calculated using CompuSyn software (ComboSyn, Inc., Paramus, NJ, USA). CI < 1 indicates synergy, CI = 1 indicates additivity, and CI > 1 indicates antagonism 36.

### Nude mice xenograft tumor

Four-week-old female BALB/c nude mice from Shanghai Jihui experimental animal were divided into groups as indicated and subcutaneously injected with 5 × 10^6^ MDA-MB-231 cells per mouse. Tumor volume, calculated as (length × width²)/2 (mm³), and tumor weight were assessed every third day. When the tumor volume reached 150 mm³, the mice were intraperitoneally injected with SSD 20 mg/kg, or irradiated with 8 Gy, or drug injection followed by irradiation. For MRE11 function analysis, nude mice were divided into 3 groups and subcutaneously injected with 5 × 10^6^ MDA-MB-231 cells expressing either a vector control, wild-type MRE11, or MRE11 mutant (K673R). The experiment was terminated when the biggest tumor reached 1 cm in length, and the mice were euthanized with sodium pentobarbital at 100 mg/kg. Tumors were harvested, measured, weighed, and paraffin-embedded for sectioning and histological analyses (TUNEL, Ki67, and γH2AX IHC). Major organs (heart, liver, spleen, kidneys) were sectioned and H&E-stained.

### TNBC tissue microarrays and immunohistochemistry (IHC)

TNBC tissue microarrays approved by the Ethics Committee of Shanghai Outdo Biotech Co.,Ltd (No.22005DKA21300) were utilized to analyze expression levels of HDAC5 using Anti-HDAC5 antibodies (Cat#16166-1-AP, 1:400, Proteintech). After the corresponding secondary antibodies incubation (Cat#111-035-003, 1:400, Jackson) and 3,3′-diaminobenzidine (DAB) color development, images were acquired using an Aperio XT scanner (Leica), and HDAC5 staining scores were calculated based on percentage of positive area and staining intensity.

### Transcriptome sequencing and TCGA data analysis

Total RNA was isolated from MDA-MB-468 cells treated with or without 7.5 μM SSD using Trizol reagent. For transcriptome sequencing, untreated cells and cells treated with lactate or sodium oxamate were processed in triplicate (1 × 10⁷ cells per replicate). Following mRNA enrichment via oligo (dT) magnetic beads, RNA was fragmented (~300 bp) and converted into sequencing libraries through PCR amplification. Libraries were sequenced on an Illumina HiSeq platform (second-generation sequencing). Transcript annotation utilized Ensembl database references. RSEM quantified gene expression, and DESeq2 identified differentially expressed genes. Functional enrichment (GO and KEGG) was analyzed using R. Gene expression validation employed real-time PCR.

Transcriptomic data for HDAC5 in TNBC samples were retrieved from the TCGA database (http://xena.ucsc.edu/) and analyzed to compare expression levels between tumor tissues and adjacent normal tissues.

### Proteomics-MS analysis

Proteomics-MS analysis was performed by Hangzhou Cosmos Wisdom (Zhejiang, China). MDA-MB-468 cells in 10 cm dishes were treated with SSD for 24 h or left as untreated controls. Cell lysates (8 M urea + protease inhibitors) were processed through: 1) reduction with 5 mM DTT (30 min), 2) alkylation with 15 mM iodoacetamide (25 °C, 15 min), 3) acetone precipitation, and 4) tryptic digestion (1:50 w/w, 100 mM TEAB, 37 °C, overnight). LC-MS/MS analysis followed (Orbitrap Astral-MS, Thermo Scientific). Data were processed by DIA-NN 1.8.1, followed by bioinformatics analyses (differential expression protein, functional classification, KEGG, etc.).

### Chromatin immunoprecipitation-quantitative polymerase chain reaction (ChIP-qPCR)

ChIP assays were performed using the Magna ChIP G Assay kit (Cat#26156, Thermo Fisher). Cells were cross-linked with 1% formaldehyde at room temperature for 10 min, followed by sonication to fragment chromatin. In the cell lysate, 10 μg of HIF1α antibody (Cat#20960-1, Proteintech) was added with protein A/G magnetic beads and incubated overnight at 4 °C. The target protein-DNA complexes were captured, and for each immunoprecipitation (including input control), 150 µL of 1 × IP elution buffer and proteinase K were mixed and incubated at 65 °C for 1.5 h. Following column-based DNA purification, qPCR was performed targeting predicted HDAC5/HIF1α binding sites; primer sequences are provided in Supplementary Table S1.

### Promoter luciferase reporter gene activity assay

Based on JASPAR database predictions of potential transcription factor binding sites within the -2 kb upstream region of the HDAC5 promoter, four truncated mutant sequences (HDAC5 #1 to #4) were designed and cloned into the pGL3-Basic vector to generate transfection plasmids. One day prior to transfection, cells were seeded in 24-well plates to achieve 60%-70% confluence. Transfection complexes were prepared by diluting 0.5 µg/µL plasmid DNA with Lipofectamine™ 2000 in Opti-MEM medium, followed by a 20-min incubation. The mixture was then added to cell cultures. 4 h post-transfection, the medium was replaced with DMEM, and cells were cultured for an additional 28 h. Subsequently, cells were treated with SSD-containing medium (7.5 μM) for 8 h before harvest for dual-luciferase assay. Firefly luciferase activity was measured and normalized to Renilla luciferase (internal control) using the DLR assay System (Promega). Each plasmid construct was transfected in three independent biological replicates.

### Molecular docking

For MRE11 and HDAC5 molecular docking, the protein structure of MRE11 (AF-P49959-F1-model_v4) and HDAC5(AF-K7EJZ7-F1-v4) were downloaded from the AlphaFold database (https://alphafold.com/) 37, 38. Protein-protein docking was performed using the HADDOCK (http://hdock.phys.hust.edu.cn/) with default parameters 39, and results were visualized using PyMOL 2.6.0 40.

### Statistical analysis

Three independent replicates (≥ 5 random IHC fields/sample) were evaluated for each experiment. Continuous data reported as mean ± SD. Group comparisons used: Student's t-test (2 groups) or ANOVA and nonparametric Kruskal-Wallis test (≥3 groups) implemented in GraphPad Prism 8.0 (GraphPad Software). *P* values <0.05 were significant.

## Results

### Lactate as a critical inducer in the development of radioresistance

**Figure 1:**
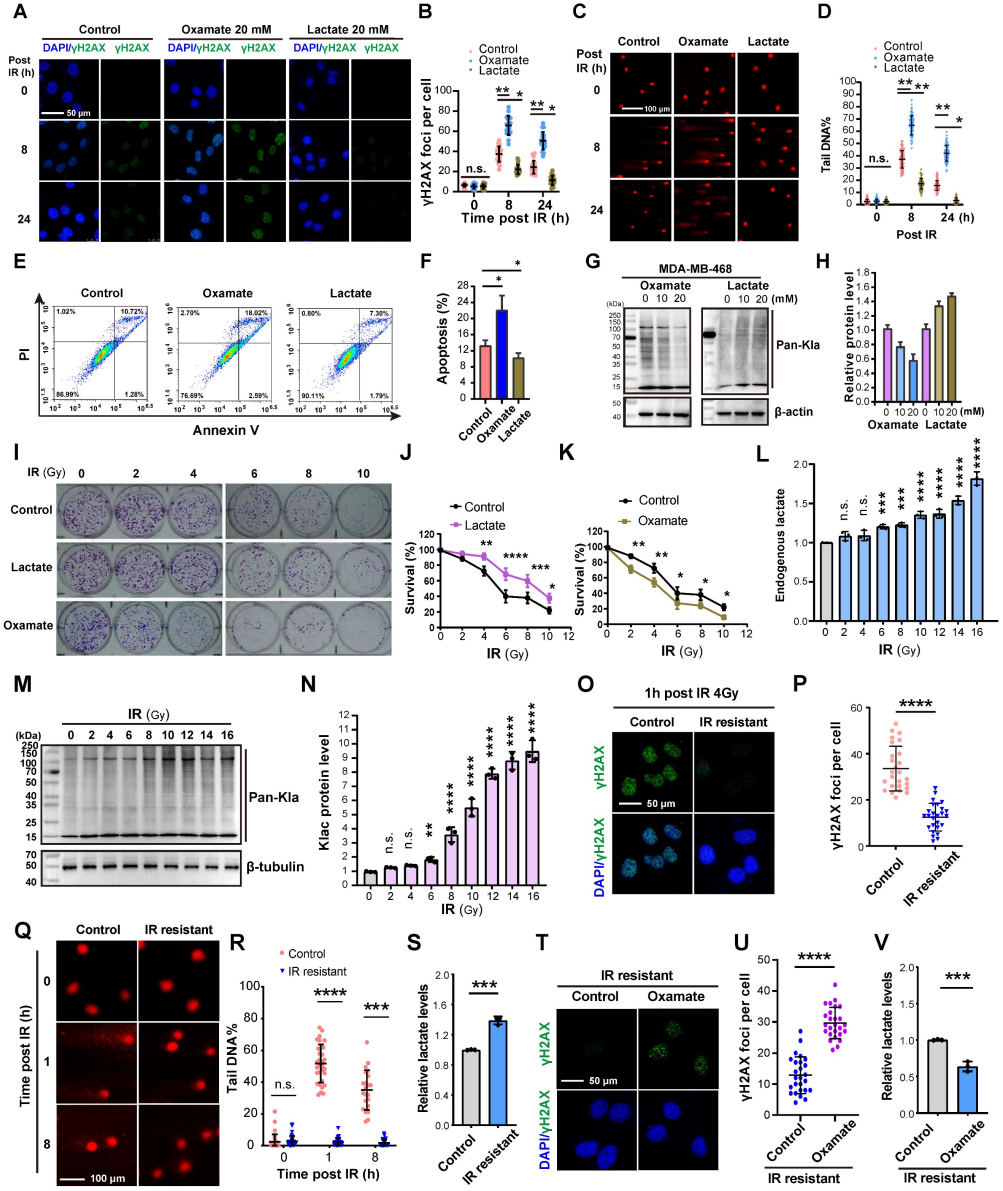
** Lactate promotes the repair of DNA damage caused by radiotherapy.** (A-B) Cells pretreated with Oxamate and lactate for 24 h were irradiated with 4 Gy, and γH2AX foci levels were observed by immunofluorescence at 8h and 24h. IR, irradiation. (C-D) Cells pretreated with Oxamate and lactate for 24 h were irradiated with 4 Gy, and the DNA damage repair ability was detected by comet electrophoresis. (E-F) Cells pretreated with Oxamate and lactate for 24 h were irradiated with 4 Gy, and apoptosis was detected by flow cytometry. (G-H) MDA-MB-468 Cells pretreated with Oxamate and lactate for 24 h were irradiated with 4 Gy, and pan-lactylation levels were detected by Western blot using a pan-lactylation antibody. (I-K) Cells were treated with multiple small doses of radiotherapy (2 Gy per session) for 0-10 sessions. The effects of lactate and Oxamate pretreatment on cell proliferation were assessed by colony formation assay. (L) Endogenous lactate levels were measured. (M-N) WB analysis of pan-lactylation protein. (O-P) γH2AX immunofluorescence were examined in IR resistant or control cells 1 h post 16 Gy of radiation. (Q-S) comet tail and endogenous lactate were detected in in IR resistant or control cells at indicated time post 16 Gy of radiation. (T-V) IR resistant strains pretreated with Oxamate followed by 4 Gy irradiation, γH2AX immunofluorescence and endogenous lactate were detected.

We utilized Oxamate, an inhibitor of intracellular lactate production, to explore its role in DNA damage repair. Treatment with 20 mM Oxamate for 24 h effectively reduced lactate production in TNBC cell lines MDA-MB-231 and MDA-MB-468 (Figure S1A). Immunofluorescence analysis of γH2AX (a marker of DNA double-strand breaks (DSBs) caused by ionizing radiation, phosphorylated on Ser-140 and dephosphorylated upon DNA repair) revealed a significant increase in γH2AX foci in Oxamate-pretreated cells after 4 Gy irradiation compared to controls (Figure 1A-B), indicating impaired DNA repair capacity. Comet assay further confirmed that Oxamate extended comet tail lengths, reflecting unrepaired DNA fragments (Figure 1C-D), which correlated with enhanced apoptosis (Figure 1E-F). Conversely, pretreatment with 20 mM lactate for 24 h followed by 4 Gy irradiation reduced γH2AX expression, shortened comet tails, and decreased apoptosis, demonstrating lactate-mediated promotion of DNA repair (Figure 1C-F). Consistent with our previous work 35, Oxamate pretreatment reduced protein pan-lactylation, while exogenous lactate increased lactylation levels (Figure 1G-H; Figure S1B-C).

Following, to mimic clinical radiotherapy conditions, a fractionated irradiation protocol (2 Gy per session) was employed instead of a single high-dose approach. Colony formation assays after 0-10 irradiation sessions demonstrated distinct effects of lactate and Oxamate pretreatment: lactate significantly promoted cell proliferation (contributing to resistance), whereas Oxamate suppressed proliferation (enhancing sensitivity) (Figure 1I-K). Cells exposed to cumulative radiation doses of 6-16 Gy exhibited a substantial increase in endogenous lactate levels (Figure 1L) and protein pan-lactylation (Figure 1M). Notably, cells receiving 16 Gy total irradiation showed undetectable γH2AX foci and comet tails upon subsequent irradiation, indicative of established radioresistance (Figure 1Q-R), accompanied by markedly elevated endogenous lactate (Figure 1S). Remarkably, Oxamate pretreatment of radioresistant cells restored their susceptibility to radiation, as evidenced by the reappearance of DNA damage markers following a 4 Gy dose radiation (Figure 1T-V). These results highlight lactate as a critical inducer in the development of radioresistance.

### Lactylation of MRE11 at K673 in response to lactate stimulates radioresistance

**Figure 2:**
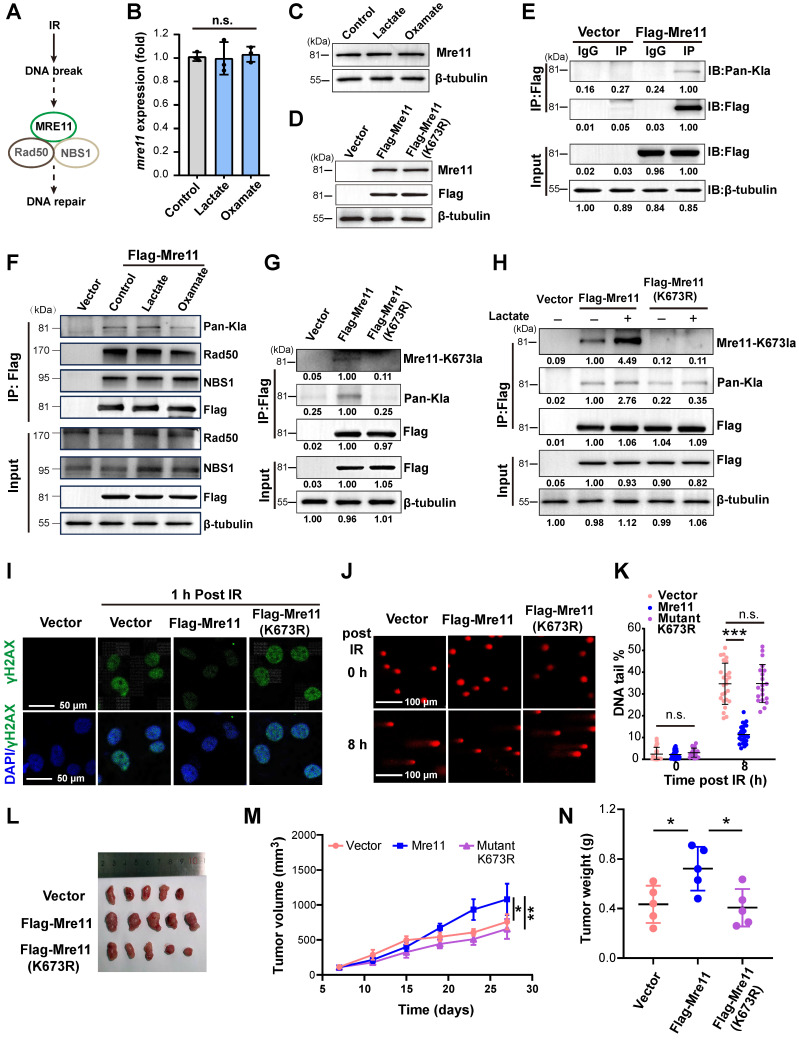
** Lactylation of MRE11 at K673 in response to lactate stimulates radioresistance.** (A) The MRN complex, composed of MRE11-Rad50-NBS1, involving in the repair of DNA double-strand breaks (DSBs). (B-C) Real-time PCR and WB analyses of MRE11 expression in cells exposed to lactate and Oxamate treatment. (D) A lentiviral overexpression vector of flag-MRE11 was constructed and transfection, and WB was used to verify expression. (E) Co-IP and WB were performed to detect whether lactylation modifications exist on Mre11, with vector and IgG as controls. (F) Co-IP and WB analysis of Mre11 lactylation levels regulated by lactate and Oxamate. (G) A Flag-Mre11 mutant was constructed, and compared to the wild-type Flag-Mre11, endogenous lactate caused significantly higher MRE11 K673 lactylation and pan-lactylation in the wild-type than in the mutant. (H) Under lactate stimulation, MRE11 K673 lactylation level of wild-type Flag-Mre11 significantly increased compared to the mutant and empty vector groups. (I-K) The effects of wild-type and mutant Flag-Mre11 on γH2AX and comet tail assays were assessed. (L-N) MDA-MB-231 cells expressing empty vector, wild-type Flag-Mre11, or mutant Flag-Mre11 were subcutaneously implanted in mice, and tumor volume and weight were measured.

Radiation-induced DNA damage triggers the cell's repair mechanisms, with the MRN complex (MRE11-RAD50-NBS1) playing a key role in resolving DNA double-strand breaks (DSBs)—the most serious DNA damage (Figure 2A). As the core component of the MRN, MRE11 is involved in DSB repair, DNA recombination, telomere integrity maintenance, and meiosis. To investigate whether lactate-induced radioresistance is associated with MRE11, we first assessed its expression under lactate treatment. Neither mRNA nor protein levels of MRE11 showed evident changes (Figure 2B-C), prompting us to construct a lentiviral vector for MRE11 overexpression to explore amino acid modifications (Figure 2D). Co-IP with a Flag antibody followed by WB with a pan-lactylation antibody revealed lactylation of MRE11 (Figure 2E), with lactylation levels dynamically regulated by lactate and Oxamate (Figure 2F). However, MRE11 lactylation did not influence the protein combination of MRN complex.

Since lactylation occurs on lysine residues 9, and lactylation of MRE11 at K673 has been shown to confer chemoresistance while other lysine sites remain unmodified 21, we sought to investigate the role of this specific site in radioresistance of TNBC. We first mutated K673 to Arginine (R) (Figure 2G). Strikingly, lactate stimulation induced significantly higher lactylation in wild-type Flag-MRE11 compared to the mutant and control groups (Figure 2H), identifying K673 as a critical lactylation site. In Flag-MRE11-overexpressing cells, DNA damage markers (γH2AX foci and comet tail lengths) were markedly reduced, whereas the mutant and control groups showed no difference (Figure 2I-K, Figure S2), confirming K673 as the key residue for lactate-induced MRE11 lactylation. *In vivo* tumor volume and weight analyses (Figure 2L-N) validated these findings, demonstrating that lactylation of MRE11 at Lys^673^ is a central mediator of radioresistance.

### HDAC5 as a key mediator of MRE11 Lys^673^ delactylation

**Figure 3:**
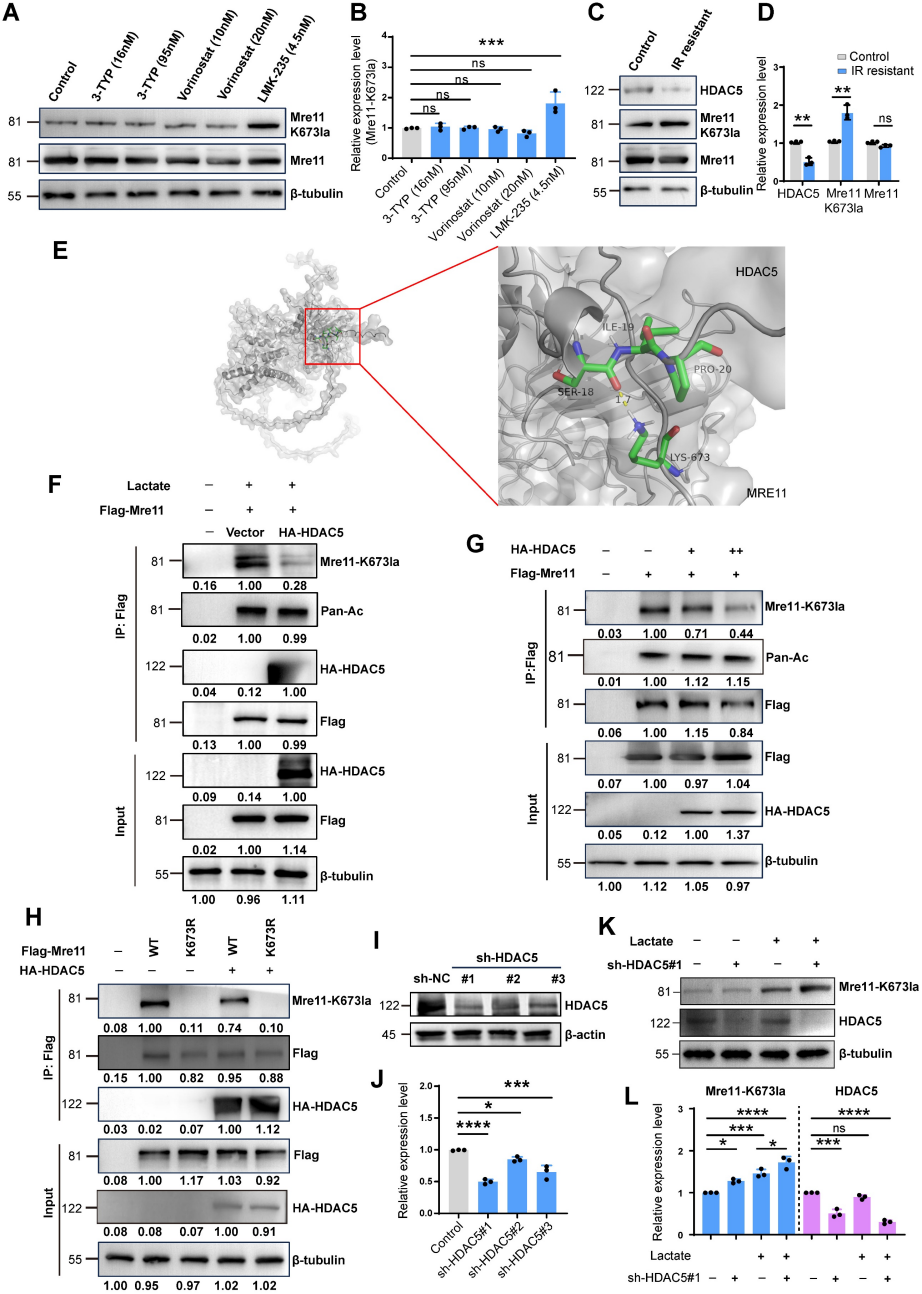
** HDAC5 plays a key role in the removal of lactylation at the K673 site of MRE11.** (A-B) Using deacetylase inhibitors including Vorinostat (selective inhibitors for HDAC1 (IC50 10 nM) and HDAC3 (IC50 20 nM), 3-TYP (SIRT1/2/3), and LMK-235 (broad-spectrum HDAC inhibitor at high doses, selective HDAC5 inhibitor at 4.2 nM), the lactylation level of Mre11 K673 were detected by WB. (C-D) WB analysis of HDAC5 and Mre11 K673 lactylation in IR-resistant cells. (E) HADDOCK molecular docking shows that Lys^673^ of MRE11 forms a hydrogen bond with SER18 of HDAC5. (F-H) Co-IP and WB analyses of overexpression of HA-HDAC5 influence on the MRE11 K673 lactylation. (I-J) WB examination of HDAC5 knockdown levels, sh-HDAC5 #1 showing the best efficiencies. (K-L) WB analysis of knockdown HDAC5 increasing MRE11 K673 lactylation under lactate stimulation.

Using deacetylase inhibitors including Vorinostat (selective for HDAC1 (IC50 10 nM) and HDAC3 (IC50 20 nM) at the concentrations used), 3-TYP (inhibitor of SIRT1/2/3), and LMK-235 (selective for HDAC5 at 4.2 nM), we found that HDAC5 inhibition enhanced MRE11 Lys673 lactylation, indicating HDAC5 as a potential lactyl-eraser (Figure 3A-B). In IR-resistant cells, reduced HDAC5 expression correlated with increased MRE11 K673 lactylation (Figure 3C-D). Subsequently, we utilized HADDOCK for docking analysis and discovered that a hydrogen bond between MRE11 K673 and HDAC5 Ser18 (Figure 3E). CO-IP results demonstrated that overexpression of HA-HDAC5 led to a significant reduction in lactylation at the MRE11 K673 site (Figure 3F). Furthermore, as the expression level of HA-HDAC5 increased, there was a clear decrease observed for MRE11 K673 (Figure 3G-H). Finally, shRNA-mediated HDAC5 knockdown efficiently silenced HDAC5, with sh-HDAC5 #1 showing the highest knockdown efficiency (Figure 3I-J). Following HDAC5 downregulation, MRE11 K673 lactylation increased significantly (Figure 3K-L). Collectively, these findings confirm HDAC5 as a key regulator of MRE11 Lys^673^ delactylation.

### Low expression of HDAC5 in TNBC can be upregulated by SSD

**Figure 4:**
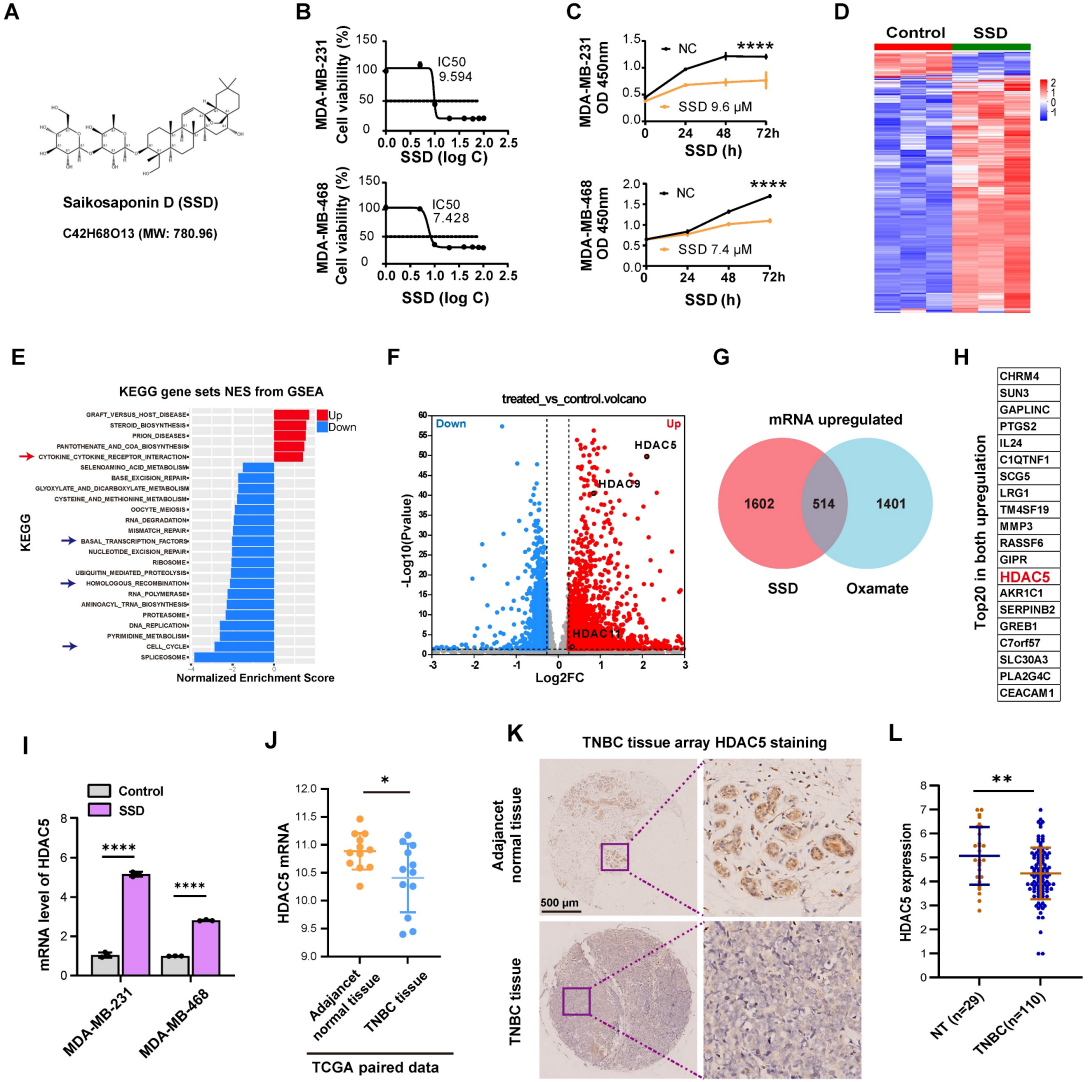
** HDAC5 expression is low in TNBC and can be upregulated by SSD.** (A) The chemical structural formula of SSD. (B-C) The inhibitory effects of the drug SSD on MDA-MB-231 and -468 cells were assessed using the CCK8 assay. (D) MDA-MB-468 cells were treated with SSD, and gene expression profiling was analyzed. (E) KEGG pathway analysis (gene sets NES from GSEA). (F) A volcano plot shows that HDAC5 is significantly upregulated. (G) The upregulated genes from SSD and Oxamate treatment were intersected, resulting in 514 common upregulated genes. (H) A list of the top 20 common upregulated genes, including HDAC5, is shown. (I) Real-time PCR analysis of HDAC family genes in SSD-treated MDA-MB-231 and -468 cells. (J) TCGA database analysis of paired TNBC data revealed that HDAC5 expression lowered in cancer tissues compared to adjacent normal tissues. (K-L) Clinical TNBC tissue microarrays were analyzed by immunohistochemistry, showing low expression of HDAC5 in tumor tissues.

SSD, an active component of Bupleurum (Figure 4A), exhibits potent antitumor activity 41, though its effects on TNBC remain underexplored. We found SSD inhibited MDA-MB-231 and MDA-MB-468 cell growth with IC_50_ values of 9.594 µM and 7.428 µM, respectively (Figure 4B-C). To dissect its mechanism, gene expression profiling revealed SSD altered cell cycle and DNA damage repair pathways in MDA-MB-468 cells (Figure 4D-E), with HDAC5 emerging as a top-upregulated gene in volcano plot analysis (Figure 4F). Given SSD and Oxamate both enhance radiosensitivity, we identified 514 genes commonly upregulated by both, with HDAC5 ranking in the top 20 (Figure 4G-H; Figure S3). RT-qPCR validated SSD-specific HDAC5 upregulation in both cell lines, without affecting other HDAC subtypes (Figure 4I, Figure S4). Furthermore, we downloaded paired data from the TCGA database for TNBC tissues and found that HDAC5 expression was lower in cancer tissues compared to adjacent normal tissues (Figure 4J), providing big data support for the upregulation of HDAC5 by SSD. Further, clinical TNBC tissue microarray staining showed that HDAC5 localized to normal breast nuclei but was downregulated in cancer tissues (Figure 4K-L). Collectively, these data demonstrate that SSD-mediated HDAC5 upregulation in cell lines may rescue low HDAC5 expression in TNBC, thus offering a therapeutic strategy for TNBC.

### HDAC5-mediated MRE11 de-lactylation underlies SSD's antitumor activity in TNBC

**Figure 5:**
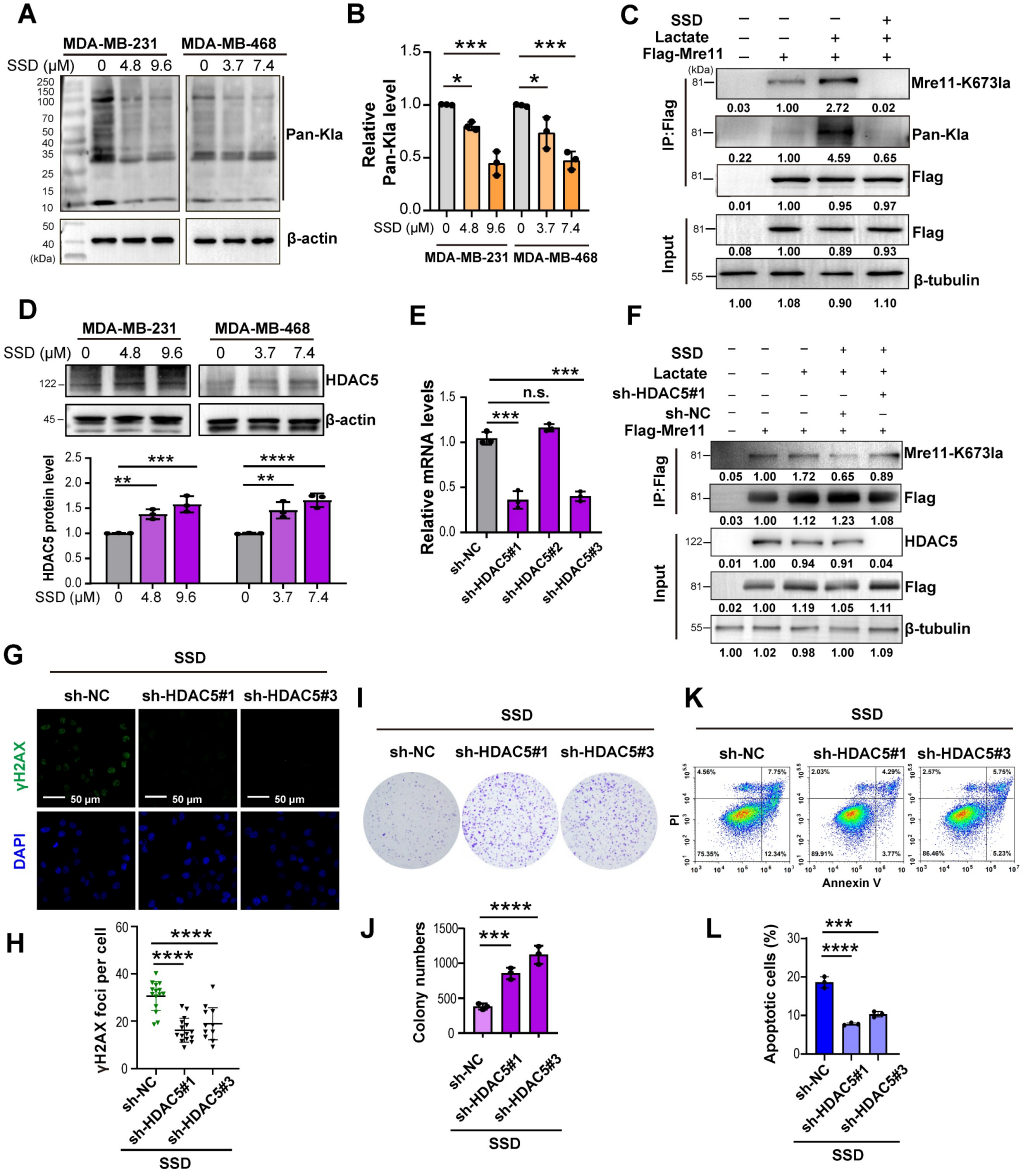
** HDAC5-mediated MRE11 de-lactylation underlies SSD's antitumor activity in TNBC.** (A-B) WB analysis of the effect of SSD on the expression of pan-lactylation proteins in cells. (C) Cells pretreated with SSD for 24 h and then stimulated with lactate were analyzed by WB for MRE11 lactylation. (D) WB analysis of the effect of SSD treatment on HDAC5 protein expression. (E) Knockdown efficiency of HDAC5 was verified by real-time PCR. (F) Co-IP and WB analysis of the effect of HA-HDAC5 transfection on lactate-induced MRE11 K673 lactylation. (G-L) The effects of HDAC5 knockdown on DNA damage, apoptosis, and cell survival capacity were assessed.

To elucidate the mechanistic relationship between SSD, HDAC5, and lactylation. we performed western blotting, revealing that SSD treatment dose-dependently reduced global protein lactylation (Figure 5A-B) and specifically inhibited lactate-induced MRE11 lactylation following 24 h pretreatment (Figure 5C). Additionally, SSD treatment promoted the expresion of HDAC5 protein in a dose-dependent manner (Figure 5D). Subsequently, knockdown of HDAC5 using sh-HDAC5#1 and #3 (validated for ≥ 70% efficiency, Figure 5E) showed that HDAC5 knockdown restored MRE11 K673 lactylation (Figure 5F). Furthermore, HDAC5 knockdown reversed SSD's effects on DNA damage marker γH2AX, cloning formation and apoptosis (Figure 5G-K). These findings suggest that HDAC5 plays a critical role in mediating MRE11 de-lactylation underlies SSD's antitumor activity in TNBC.

### SSD pretreatment enhances the therapeutic effect of radiotherapy

**Figure 6:**
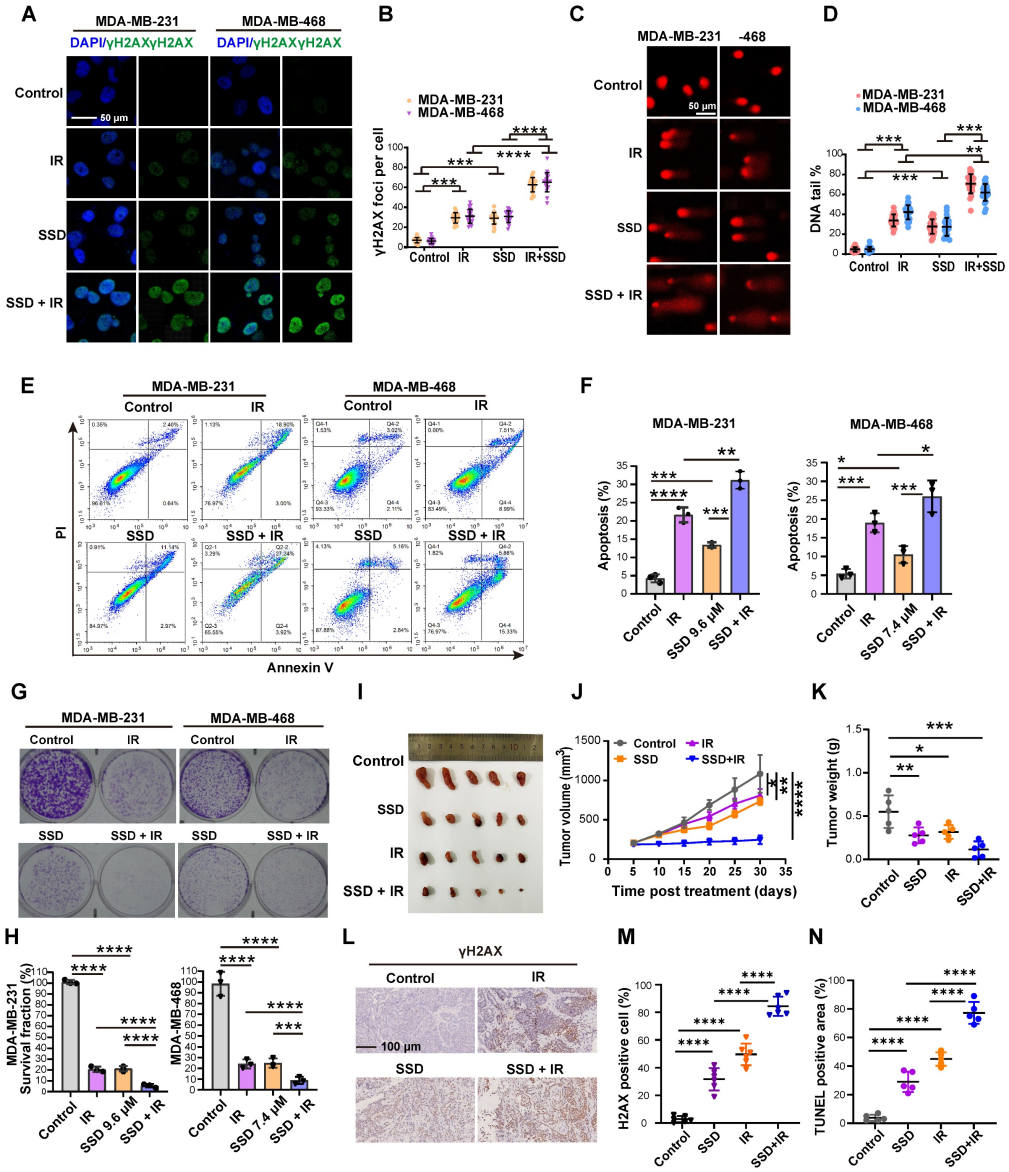
** SSD pretreatment enhances the therapeutic effect of radiotherapy.** (A-D) Cells opposed to irradiation, SSD, or combined treatment were analyzed for γH2AX and comet tail assays. (E-F) Cell apoptosis levels were measured. (G-H) Colony formation assays were conducted to assess cell proliferation. (I-K) MDA-MB-231 cells were implanted subcutaneously into nude mice and treated with SSD, irradiation 8 Gy, or SSD treatment followed by irradiation. Volume and weight were calculated. (L-N) γH2AX immunostaining and TUNEL assay.

SSD monotherapy and radiotherapy alone induced DNA damage in TNBC cells (Figure 6A-D). Notably, combinatorial treatment—24 h SSD pretreatment followed by irradiation—significantly enhanced DNA damage compared to single treatment, indicating SSD's radiosensitizing effect. This was confirmed by enhanced apoptosis (Figure 6E-F) and reduced clonogenic survival (Figure 6G-H). The combination index (CI) value from the synergy analysis was less than 1, indicating a synergistic effect between SSD and IR (Figure S5). *In vivo* studies using MDA-MB-231 xenografts showed that while SSD or irradiation monotherapy modestly inhibited tumor growth (Figure 6I-K), their combination potently enhanced radiotherapy response. IHC of γH2AX and TUNEL analyses validated increased DNA damage and apoptosis in combined treatment groups (Figure 6L-N). In addition, histopathological analysis of major organs (heart, liver, spleen, kidney) revealed no treatment-related toxicity at the present doses of SSD (Figure S6). Collectively, our findings show that SSD pretreatment potently improves radiotherapy efficacy, yet the underlying molecular mechanism remains to be elucidated.

### SSD activates HDAC5 transcription via HIF1α signaling

**Figure 7:**
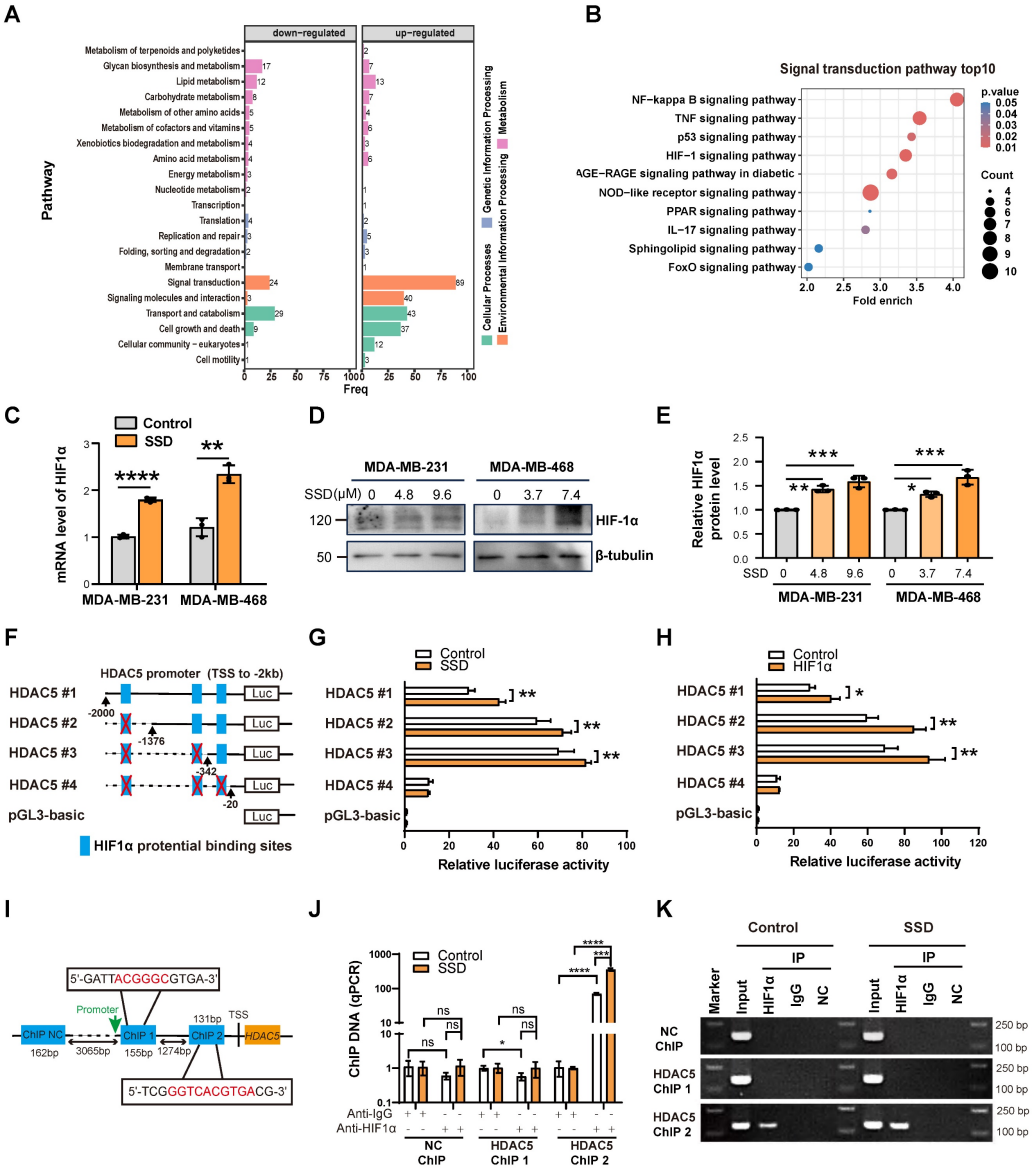
** SSD activates HDAC5 transcription via HIF1α signaling.** (A-B) Proteomic analysis of SSD influence on protein changes, showing significant alterations in signal transduction pathways, with notable activation of the HIF-1 pathways. (C-E) Realtime PCR and WB analyses of HIF1α expression in SSD-treated MDA-MB-231 and -468 cells. (F) Truncated mutant HDAC5-#1~4 according to JASPAR database potential binding sites for HIF1α within the -2kb region of the HDAC5 promoter were cloned into pGL3 luciferase reporter vectors. (G-H) Luciferase assays of SSD treatment or HIF1α overexpression effect on HDAC5 promoter activity. (I-K) ChIP-qPCR analysis revealed that SSD stimulation obtained a higher yield of HDAC5 promoter DNA located to ChIP2 primers amplified region DNA.

To investigate SSD-mediated HDAC5 upregulation, we performed proteomic-MS on MDA-MB-468 cells treated with SSD for 24h, identifying activated NF-kappaB, TNF and HIF-1 signaling (Figure 7A-B). SSD treatment increased HIF1α expression on both mRNA and protein levels (Figure 7C-E). JASPAR analysis predicted three putative HIF1α binding sites within the HDAC5 promoter -2kb region (relative to the transcription start site). Luciferase assays with truncated HDAC5 constructs (HDAC5-#1~4; -2000bp to -20bp) showed maximal promoter activity between -342bp and -20bp following SSD treatment (Figure 7F-G). Co-transfection of MDA-MB-468 cells with HDAC5 luciferase reporters and an HIF1α-overexpression plasmid further revealed that HIF1α overexpression enhanced HDAC5 promoter activity, with the highest activity notably observed between -342bp and -20bp (Figure 7H). These results indicate that SSD regulates HDAC5 promoter activity via the HIF1α pathway.

ChIP-qPCR analysis showed that HIF1α antibody enrichment with HDAC5 ChIP2 primers amplified HDAC5 promoter DNA, with the SSD-treated group yielding significantly more product than the negative control (NC) group (Figure 7I-J). Agarose gel electrophoresis of qPCR products further confirmed these results, and the ChIP2 amplification region—consistent with luciferase reporter data—spanned -342 to -20 bp (motif: ggtcacgtga) relative to the transcription start site, indicating the HIF1α binding the locus within this region (Figure 7K).

**Figure 8:**
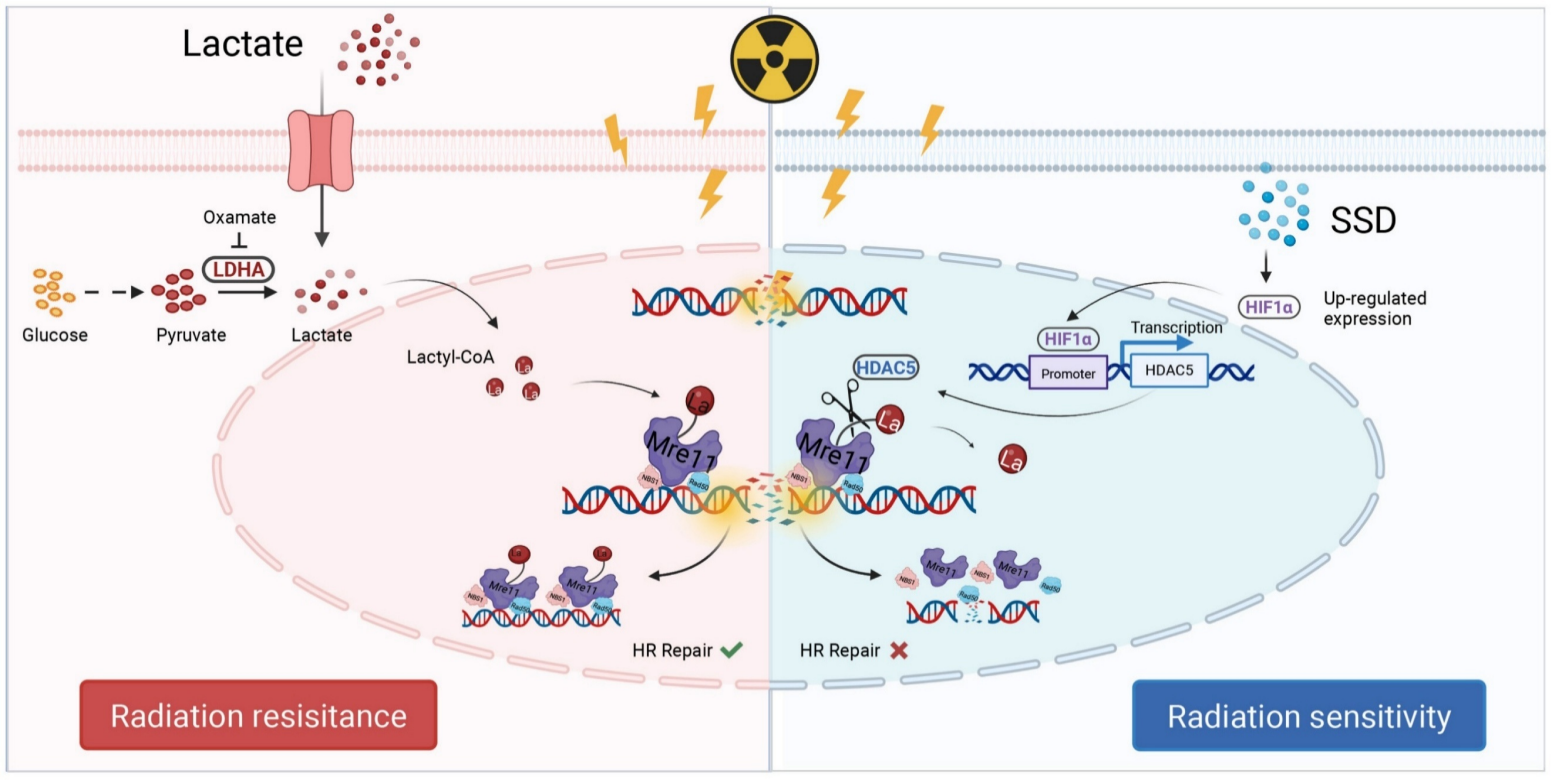
** Schematic illustration.** Lactate induces MRE11 Lys^673^ lactylation resulting in radioresistance, while SSD upregulates HDAC5 via HIF1α pathway to remove lactyl groups, thereby sensitizing TNBC cells to radiotherapy.

In summary, SSD activates the HIF1α signaling pathway (Figure 8). Subsequently, HIF1α binds to specific sites within the -342 bp to -20 bp region of the HDAC5 promoter, enhancing its transcriptional activation. Finally, HDAC5 upregulation enhances its delactylase activity toward MRE11, thereby reversing radioresistance in TNBC.

## Discussion

Radiation sensitivity is central to radiotherapy efficacy. This study reveals that lactate accumulation in the tumor microenvironment drives MRE11 K673 lactylation, promoting MRN complex-mediated homologous recombination repair and radioresistance. We show that SSD inhibits TNBC growth and synergizes with radiotherapy to induce DNA damage and apoptosis. Mechanistically, SSD promotes HDAC5 transcription via HIF1α. HDAC5 de-lactylates MRE11 K673, thereby enhancing radiosensitivity. Our results establish HDAC5 as a key mediator of MRE11 de-lactylation, potentially serving as a lactyl-eraser enzyme in TNBC.

Lactate, a key glycolytic metabolite in tumor cells, has drawn growing attention for its role in the Warburg effect 7, 42. Both chemotherapy and radiotherapy can trigger metabolic reprogramming in tumor cells by altering microenvironmental factors like nutrients and cytokines, which in turn affects therapy sensitivity. Elevated glycolysis with lactate accumulation confers resistance to chemotherapeutics including 5-fluorouracil, doxorubicin, and platinum agents 43-45. In cervical cancer, lactate upregulates histone deacetylases (HDACs) to enhance DNA repair and counter cisplatin toxicity 46. Similarly, radiotherapy promotes glycolysis and lactate secretion in pancreatic cancer cells 47. Here, we modeled clinical fractionated radiotherapy in TNBC and found that increasing radiation doses promoted lactate production. Treatment with Oxamate reversed radioresistance, confirming lactate's important role in this process.

Radiotherapy and chemotherapy induce DNA damage—either directly or indirectly—compromising genomic stability and triggering cell death. MRE11, a core subunit of the MRN complex, is central to DNA damage repair. Emerging evidence highlights MRE11 as a promising biomarker for radiosensitivity strategies: elevated tumor MRE11 levels correlate with poor survival in radiotherapy patients, while ionizing radiation-induced MRE11 truncation promotes radioresistance 24, 48. Notably, MRE11's role varies across cancer types, warranting further mechanistic clarification. Chen et al. recently identified MRE11 K673 lactylation as a driver of chemoresistance via mass spectrometry 21. Consistently, our co-immunoprecipitation studies reveal that MRE11 K673 lactylation is a pivotal modification mediating radioresistance in TNBC.

Previous studies have shown that acetylation and lactylation share overlapping sets of modifying enzymes. The primary de-lactylating enzymes identified to date belong to the HDAC and SIRT family members. Research has demonstrated that common deacetylases like HDAC1-3 and SIRT1-3 exhibit de-lactylating activity, with their regulatory roles in histone lactylation confirmed via fluorescent peptide labeling, overexpression, and RNAi assays 29, 30. HDACs are critical regulators of breast cancer cell growth and gene expression, positioning them as key targets for epigenetic anticancer drug design 43. Notably, our study uncovers an unrecognized "eraser" function for HDAC5 through its de-lactylation of MRE11. While direct biochemical evidence is lacking, we systematically validated the mechanism—using eraser enzyme inhibitors, molecular docking, Co-IP, and gain/loss-of-function assays—showing HDAC5 mediates de-lactylation of MRE11 at Lys^673^. These findings suggest that elevating HDAC5 levels in TNBC may represent a promising strategy to counteract MRE11 lactylation-driven radioresistance.

How to upregulate HDAC5? We identified that the natural bioactive compound SSD suppresses TNBC by potently upregulating HDAC5 via the HIF1α signaling pathway. Low HDAC5 expression in cancer cells has been linked to poor prognosis 49, 50. Using Gene Expression Profiling Sequencing, TCGA database mining, and tissue microarray staining, we confirmed HDAC5 downregulation in TNBC. Proteomic-MS analysis revealed that SSD treatment significantly elevates HIF1α signaling. Computational docking predicted three HIF1α-binding motifs within the HDAC5 proximal promoter (-2 kb), with luciferase and ChIP-qPCR assays validating direct binding to the -342/-20 bp element (motif: ggtcacgtga). Together, these data suggest that SSD activates HIF1α, promotes HDAC5 transcription, and enhances MRE11 de-lactylation, ultimately resulting in radiosensitization.

Notably, our study lacks clinical validation in patient samples to directly link HDAC5 expression with radioresistance. As a natural product, SSD holds promise for reducing radiotherapy toxicity and improving treatment tolerance; however, its efficacy against chemotherapy resistance remains untested and warrants investigation. While we focused on double-strand break repair—a critical challenge in radiotherapy—whether the SSD/HDAC5/MRE11 lactylation axis regulates other DNA damage repair pathways (e.g., base excision repair) requires exploration. Additionally, we did not account for immune microenvironment influence. Collectively, this work defines a molecular mechanism of radioresistance and identifies SSD as a potential radiosensitizer, providing experimental evidence for developing MRE11-targeted therapies, HDAC5 modulators, and SSD-derived drugs for future TNBC therapy.

## Conclusions

This study elucidates the molecular mechanism of radioresistance and identifies therapeutic targets, demonstrating that SSD activates HIF1α signaling, which in turn promotes HIF1α binding to a specific cis-acting element (HIF1α binding site, -342 bp to -20 bp) in the HDAC5 promoter to enhance transcription. This leads to HDAC5-mediated delactylation of MRE11 K673, thereby reversing radioresistance in TNBC. While current lactate/lactylation-targeted drugs primarily inhibit lactate transport or glycolysis, this work targets lactylation-modifying enzymes, highlighting the potential of natural compounds like SSD in overcoming radioresistance and paving the way for novel cancer therapeutic strategies.

## Supplementary Material

Supplementary figures and table.

## Abbreviations

TNBC: Triple-negative breast cancer
IR: irradiation
HDAC5: histone deacetylase 5
DSB: Double strand breaks
SSD: Saikosaponin D
WB: Western blotting
Co-IP: Co-immunoprecipitation
DAPI: 4,6-diamino-2-phenyl indole
IHC: Immunohistochemistry
Pan-Kla: Pan histone lysine lactylation
KEGG: Kyoto Encyclopedia of Genes and Genomes
RNA-seq: RNA sequencing
TUNEL: TdT-mediated dUTP Nick-End Labeling
HIF1α: Hypoxia-inducible factor 1-alpha
MRN complex: MRE11-RAD50-NBS1
shRNA: Short hairpin RNA
