# Supplementary material for: Saikosaponin D Mitigates Radioresistance in Triple-Negative Breast Cancer by Inducing MRE11 De-Lactylation via HIF1α/HDAC5 Pathway
Source: Theranostics. 2025 Aug 11;15(17):8935–51. doi: 10.7150/thno.113517 (PMC12439262; doi:10.7150/thno.113517)
Supplement: Supplementary file 1 — Supplementary figures and table. [file thnov15p8935s1.pdf]

Supplementary data:

Table S1. Primers used in the present research

Homo gene or promoter	forward and reverse primers
Mre11	5'-AGAGGAGGGTCTCAAAGAGGA-3'<br>5'-TGTGCTGGACCACCTTTGAT-3'
β-actin	5´-TACCTCATGAAGATCCTCACC-3´<br>5´-TTTCGTGGATGCCACAGGAC-3´
HDAC1	5′-TGACGAGTCCTATGAGGCCA-3′<br>5′-CACACTTGGCGTGTCCTTTG-3′
HDAC2	5′-AGCCACTGCCGAAGAAATGA-3′<br>5′-ACTGAACCGCCAGTTGAGAG-3′
HDAC3	5′-AGTTCTGCTCGCGTTACACA-3′<br>5′-CAGAAGCCAGAGGCCTCAAA-3′
HDAC5	5′-TCCTCTATTCCTGGCTGCCT-3′<br>5'-CACACGTTCACCCGTCACTA-3'
HDAC9	5′-CCCAACATTACCTTGGGGCT-3′<br>5'-AACATGAGGGTGGCTGGAAG-3'
HDAC11	5′-TCTGTGCCTATGCGGACATC-3′<br>5'-ACGCTTGTCGTCCATGAAGT-3'
HIF1 α	5′-GGGCAAACTTTCTGGCAGTG-3′<br>5'-TCCTGGATTCCGTGGAGGAT-3'
IFNGR1	5′-CTGCCAGGTTCAGACTGGTT-3′<br>5'-CCAGGCATGCATACCGAAGA3'
ChIP NC	5'TGGCTCATGCCTGTAATCCCAG 3'<br>5'AGTAGCTGGGACTACAGGAGCG 3'
HDAC5 Prpmpter ChIP 1	5'TGTTAGCCAGGGTGGTCTCG 3'<br>5'ACCAGCCTGGGTAACATGGC 3'
HDAC5 promoter ChIP 2	5'TCTATTTTTAGCTCCGGGTCGG 3'<br>5'GGCGGCAGCACCTCCTCGAC 3'

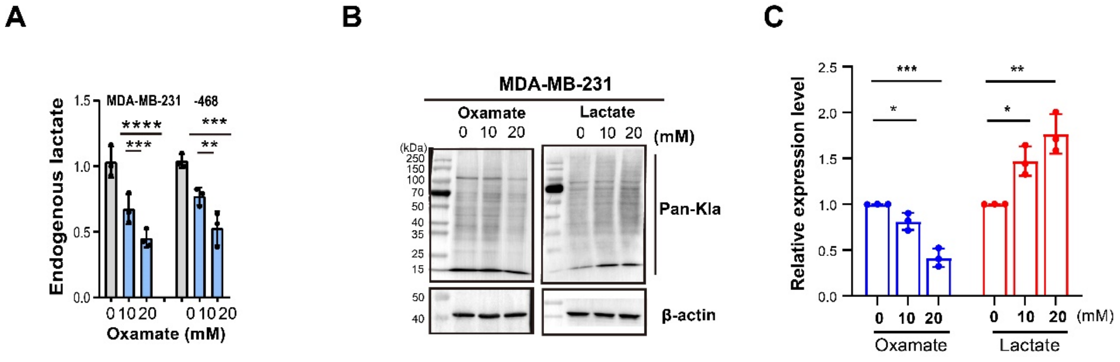

Figure S1. Detection of endogenous lactate. (A) Treatment with 20 mM Oxamate for 24 h effectively reduced lactate production in TNBC cell lines MDA-MB-231 and MDA-MB-468. (B-C) Oxamate pretreatment reduced protein pan-lactylation, while exogenous lactate increased lactylation levels in MDA-MB-231 cells.

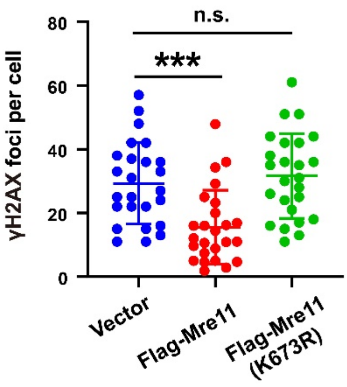

Figure S2. Immunofluorescence staining revealed significantly fewer γH2AX foci (a DNA damage marker) in Flag-MRE11-overexpressing cells, with no difference between the mutant and control groups.

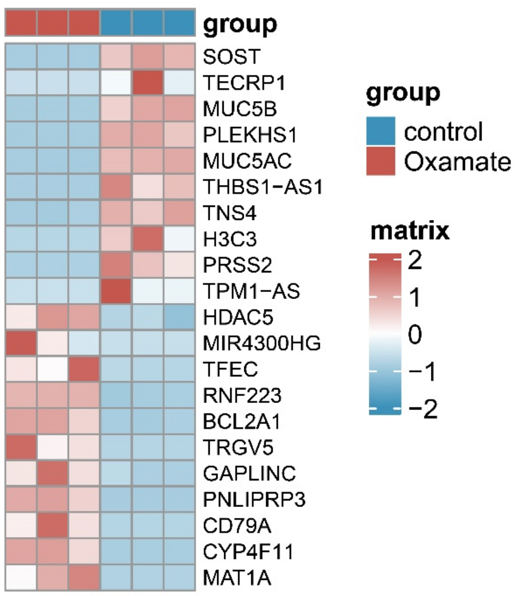

Figure S3. The heatmap from transcriptome sequencing data shows the impact of 20 mM oxamate treatment for 24h on gene expression in MDA-MB-231 cells.

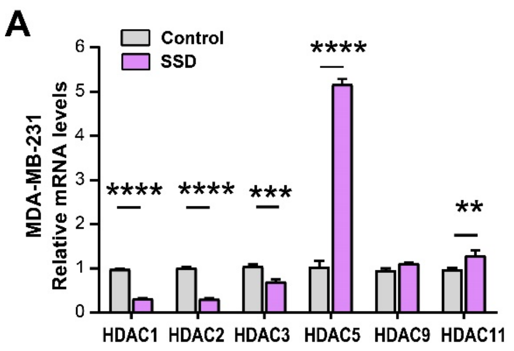

Figure S4. RT-qPCR validated SSD-specific HDAC5 upregulation in both cell lines, without affecting other HDAC subtypes.

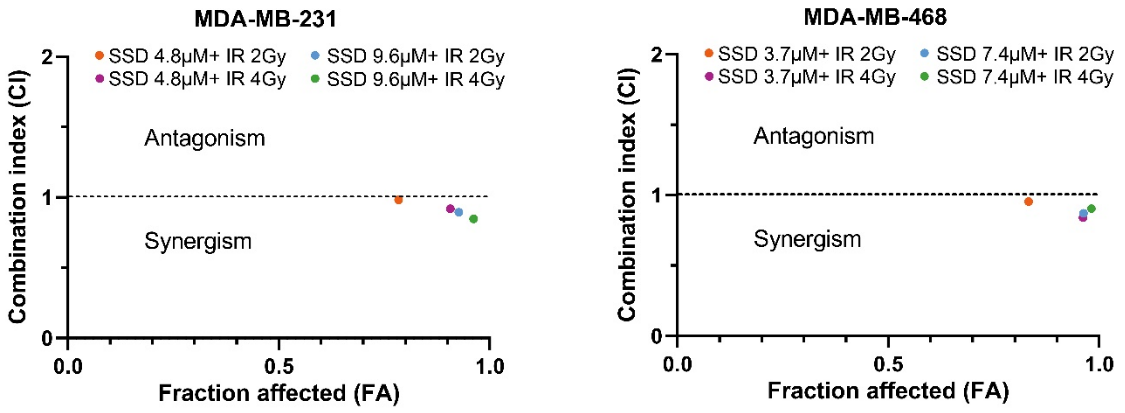

Figure S5. Synergy analysis of SSD and IR. To assess the synergistic effects between SSD and radiotherapy, triple-negative breast cancer (TNBC) cells were treated with gradient concentrations of SSD and varying radiation doses. Combination Index (CI) values for the two therapeutic agents were calculated using CompuSyn software (ComboSyn, Inc., Paramus, NJ, USA). CI values < 1 in both MDA-MB-231 and MDA-MB-468 cells indicate a synergistic interaction between SSD and IR.

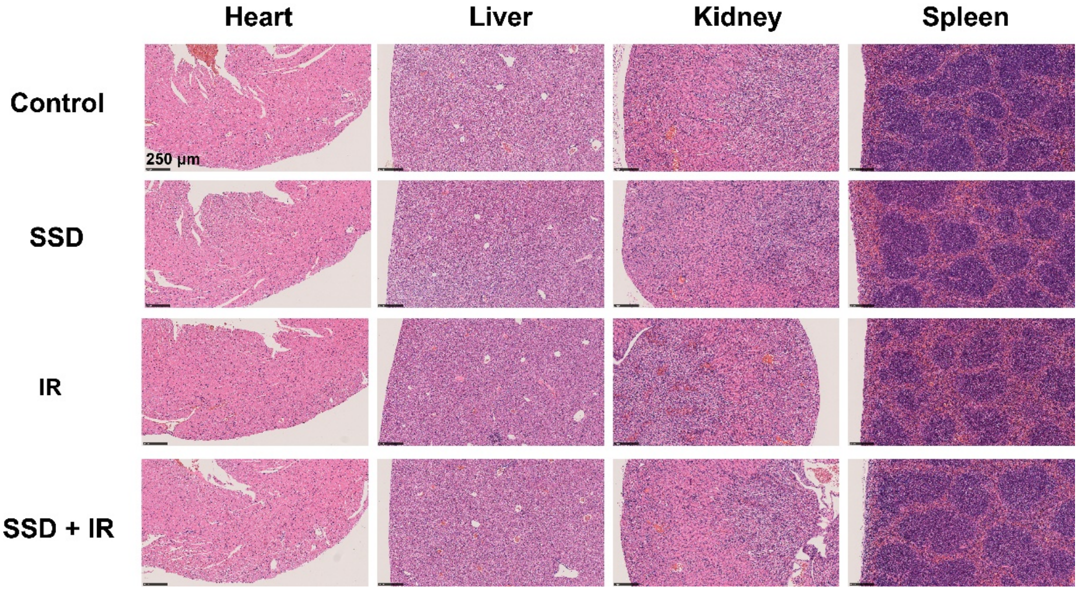

Figure S6. Histopathological analysis of major organs (heart, liver, spleen, kidney) revealed no treatment-related toxicity at the present doses of SSD.
